# Engineered Marine Biofilms for Ocean Environment Monitoring

**DOI:** 10.1021/acssynbio.5c00192

**Published:** 2025-06-23

**Authors:** Guillermo Nevot, Maria Pol Cros, Lorena Toloza, Nil Campamà-Sanz, Maria Artigues-Lleixà, Laura Aguilera, Marc Güell

**Affiliations:** † Department of Medicine and Life Sciences, 16770 Universitat Pompeu Fabra, Barcelona 08005, Spain; ‡ ICREA, Institució Catalana de Recerca i Estudis Avançats, Barcelona 08010, Spain; § Department of Cell and Molecular Biology, Karolinska Institutet, Solna 17165, Sweden

**Keywords:** ELMs, marine bacteria, *Dinoroseobacter shibae*, surface colonization, biofilm, biosensors

## Abstract

Marine bacteria offer a promising alternative for developing Engineered Living Materials (ELMs) tailored to marine applications. We engineered to increase its surface-associated growth and develop biosensors for ocean environment monitoring. By fusing the endogenous extracellular matrix amyloidogenic protein CsgA with mussel foot proteins, we significantly increased biofilm formation. Additionally, was engineered to express the tyrosinase enzyme to further enhance microbial attachment through post-translational modifications of tyrosine residues. By exploiting natural genetic resources, two environmental biosensors were created to detect temperature and oxygen. These biosensors were coupled with a CRISPR-based recording system to store transient gene expression in stable DNA arrays, enabling long-term environmental monitoring. These engineered strains highlight potential in advancing marine microbiome engineering for innovative biofilm applications, including the development of natural, self-renewing biological adhesives, environmental sensors, and “sentinel” cells equipped with CRISPR-recording technology to capture and store environmental signals.

## Graphical abstract

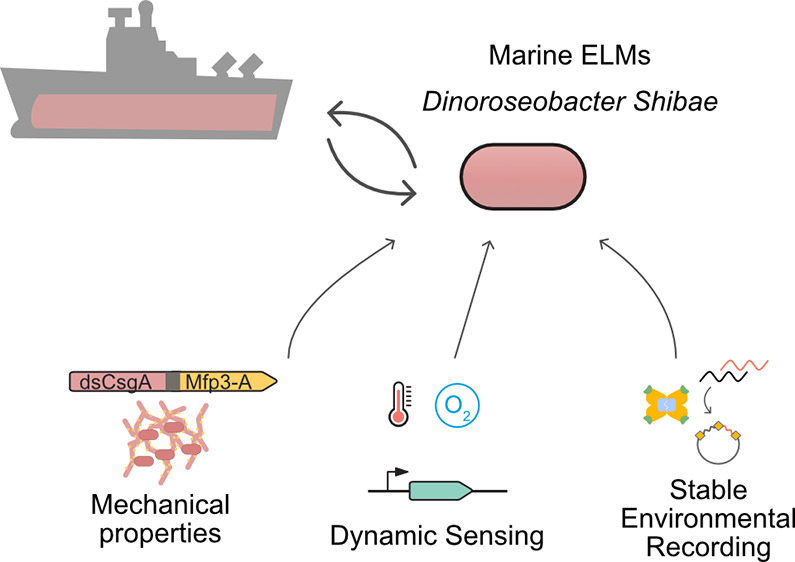

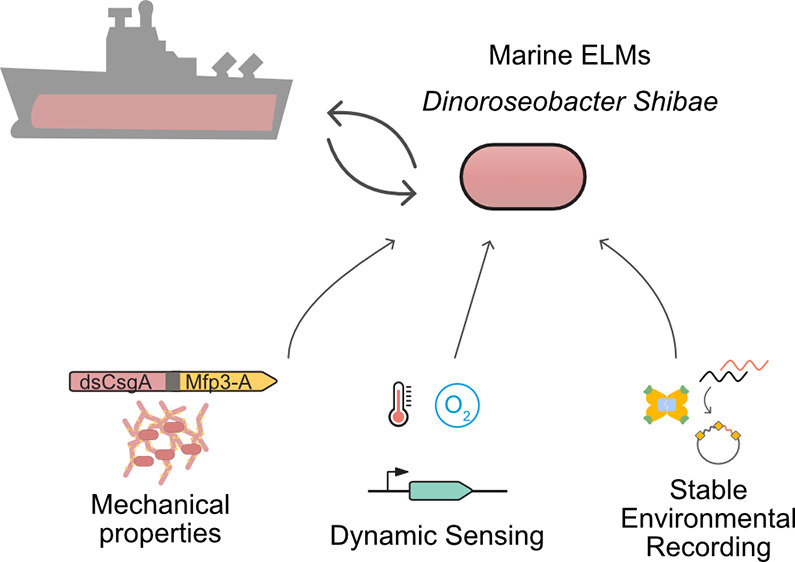

## Introduction

Synthetic biology aims to program life and enhance the natural adaptability of microbes to the environment.^1,2^ In this context, engineering the ocean microbiome has arisen as a promising strategy to enhance naturally occurring underwater biofilms and develop engineered living materials (ELMs). Bacterial biofilms are a self-assembling extracellular matrix, which often consist of amyloid fibers.^3^ Among them, curli nanofibers, formed by CsgA subunits, have been extensively engineered, for instance, for environmental remediation,^4^ underwater adhesion,^5^ and biomedical applications.^6^ However, most of these applications have been developed in model organisms, such as or , which do not grow optimally in marine conditions. Living organisms are advantageous as they can be programmed with genetically encoded sensors to monitor environmental signals or act dynamically upon certain stimuli.^4^ For these reasons, biofilm-forming marine bacteria present a promising alternative for developing ELMs tailored to marine surfaces.

Despite this potential, marine biofilms are more commonly associated with negative outcomes, particularly in the context of biofouling. Biofouling is the accumulation of microorganisms, algae, or small animals on submerged surfaces. Primary colonizers, involving diatoms and microalgae spores, attach and form a bacterial biofilm (microfouling). This biofilm serves as a suitable environment for the proliferation of macroalgae and invertebrate larvae, such as mussels or barnacles, leading to the development of a complex macroscopic community (macrofouling).^7^ This process is particularly problematic for watercraft, as these organisms induce metal corrosion and increase hydrodynamic resistance, eventually reducing the service life of ships and elevating fuel consumption. According to the US Navy, biofouling costs are estimated to be between $180 M and $260 M per year across its fleet.^8^ Although effective at preventing fouling, commercial coatings contain toxic substances, such as heavy metals, that harm marine ecosystems.^7,9^ For instance, one of the most commonly used antifouling compounds, tributyltin or TBT, was banned in 2008 due to its toxicity to aquatic ecosystems and risks to human health.^10^ Over the last 20 years, other technologies have emerged such as PPG PSX 700, a polysiloxane technology that provides the barrier protection of an epoxy resin and the UV resistance of urethane. Although it is isocyanate-free and has a reduced environmental impact due to low volatile organic compound emissions, it is not entirely harmless to the marine ecosystem.^11^

The urge for new, nontoxic, and environmentally friendly coating strategies has led to the development of bioinspired strategies.^12^ Marine bacteria-based engineered living materials (ELMs) could be designed to be adhesive, capable of colonizing surfaces of interest, self-healing, and resistant to early microbial colonization through competitive exclusion.^5^ By preventing the initial establishment of other microorganisms, these ELMs could contribute to reducing biofouling on ship hulls and other marine structures. In addition to physically occupying the hull’s niche, ELMs can be modified to actively self-regulate biofilm thickness and counteract biofilm formation from other invading organisms with strategies such as quorum quenching^13^ or biofilm dispersal proteins.^14^

 bacteria are ubiquitous in the ocean, representing between 15 and 25% of the total marine ecosystem.^15^ Members of this clade are present in both planktonic and biofilm growth and have a flexible and versatile metabolism. For this reason, they are the primary colonizers of marine surfaces, even when covered with antifouling paints.^16^ is a Gram-negative bacterium and a member of the clade, capable of performing both aerobic anoxygenic photophosphorylation and anaerobic denitrification for energy production.^17^ The genome sequence contains 4198 protein-coding genes, of which approximately 28% have no predicted function. These include genes involved in the synthesis of vitamin B_12_, aromatic compound degradation, and sulfur metabolism.^18^ Methods for genetic manipulation of the clade have been established for the development of functional studies in marine bacteria.^19,20^ In particular, replicative plasmids and antibiotic resistance cassettes have been described for . As a proof of concept, a suicide vector has even been used to study oxygen regulators in .^21^ However, this bacterium lacks more sophisticated tools, such as inducible biosensors or CRISPR-based systems, to fully realize the potential of as an emerging marine synthetic biology chassis for advanced applications.

While many bioinspired approaches emphasize isolating biocidal compounds from marine bacteria,^22^ in this study, we developed tools to use the non-model organism as a natural chassis for marine ELMs with the objective of creating smart and dynamic biofilms able to report environmental changes, self-regulate, and prevent biofouling. Toward this aim, we engineered strains capable of colonizing underwater surfaces and able to sense environmental signals and permanently record them into DNA. First, we engineered the surface and secretome of to increase preferential surface colonization by fusing its endogenous amyloidogenic protein CsgA with different mussel foot-derived adhesive proteins.^23^ The resulting fusion proteins formed functionalized amyloid fibers, enhancing biofilm development. Moreover, we further enhanced their colonization ability by cocultivating them with an engineered strain exhibiting tyrosinase activity. The tyrosinase enzyme MelC2 performs a post-translational modification on tyrosine residues of mussel foot proteins, converting them into 3,4-dihydroxyphenylalanine (DOPA),^5,24^ which binds a wide range of substrates whether they are hydrophilic, hydrophobic, inorganic, or organic. In this context, curli fibers enhance the stability of our biofilms, thereby protecting our marine ELMs from external stressors and indirectly contributing to the prevention of external colonization.^25^ At the same time, we developed two environmental sensors to detect temperature and oxygen in . Given the low portability of genetic tools from model bacteria even among strains,^26^ we developed a strategy for rapid identification of genome-encoded biosensors from transcriptome data. Finally, we adapted the CRISPR-Cas recording technology for this bacterium,^27^ successfully recording transient gene expression into stable DNA arrays to monitor bacterial exposure to temperature increases. This system allows ticker-tape-like temporal recording, which may be very attractive for environmental monitoring.

## Results

### Fusion of CsgA to Mussel Foot Proteins Increases Biofilm Formation

CsgA has been fused to mussel foot proteins to generate functionalized curli amyloid fibrils with stronger underwater adhesion in .^28^ We engineered Dshi_0598, a homologue of the extracellular matrix protein CsgA present in (dsCsgA), along with three different variants of the mussel foot protein Mfp3 (Mfp3-A,^23^ Mfp3-S,^28^ Mfp3-SP^5^), to enhance the adhesive properties of biofilms and improve colonization success. We characterized the functionality of these dsCsgA-Mpf3 fusions in for different expression levels using two promoters: the endogenous promoter from the *csgA* gene alone (P­(dsCsgA)) or the endogenous promoter preceded by the promoter *aphII* ^21^ (P­(aphII)) (Figure 1A).

**Figure 1:**
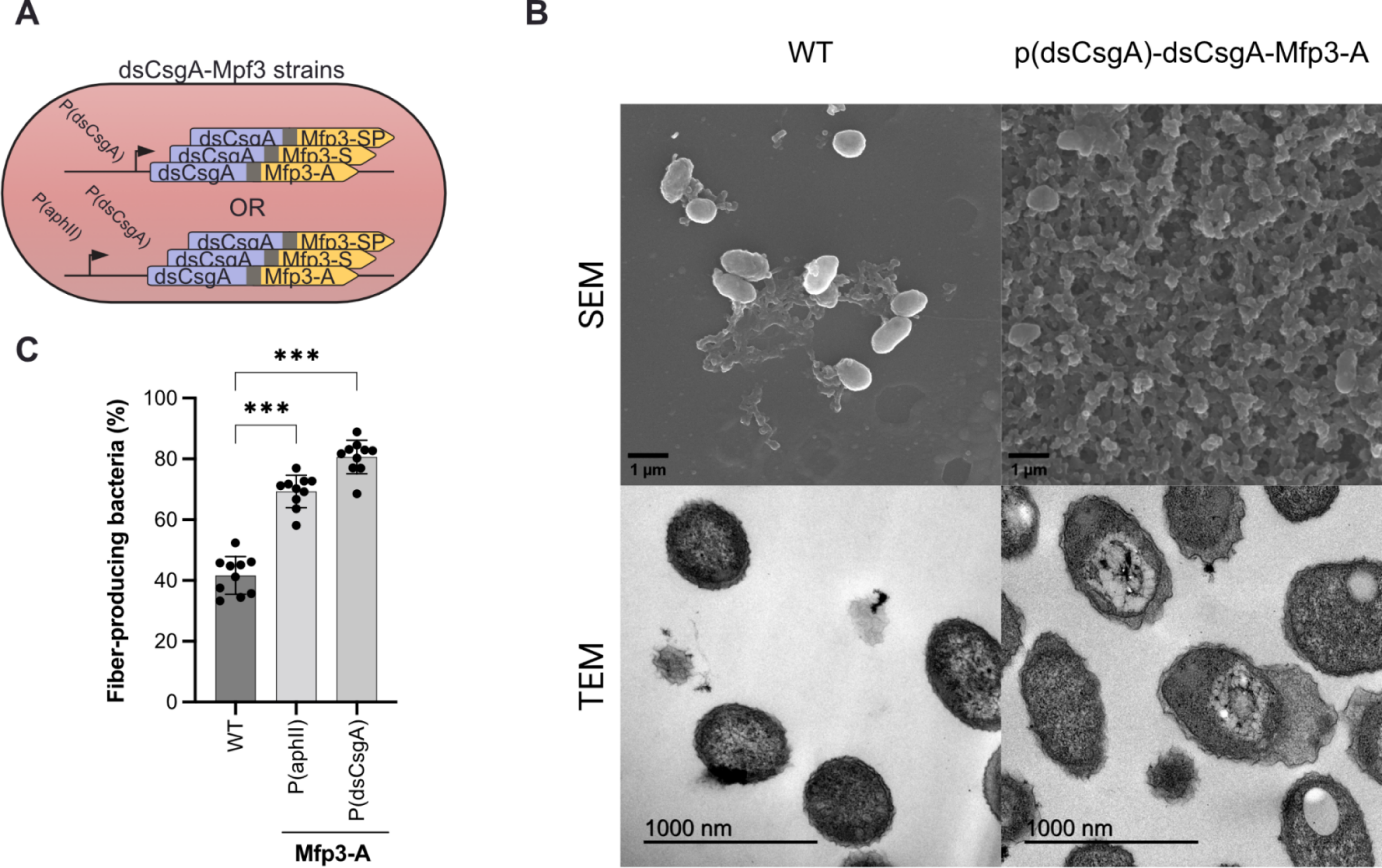
Biofilm structure in variants expressing dsCsgA and Mfp3 Genes. (A) Schematic representation of the different adhesion modules containing the dsCsgA and Mfp3 genes. (B) SEM images (first row) show an extensive fibrous network in the engineered variant, while TEM images (second row) reveal a notable fibrous network in the engineered strains. Images were taken at magnifications of 9,500× (SEM) and 60,000× (TEM). (C) Percentage of bacteria producing amyloid fibers, calculated from scanning TEM images at 20,000× magnification. Data are presented as mean ± SD (*p ≤ 0.05, **p ≤ 0.01, ***p ≤ 0.001).

To study the structure of these functionalized curli amyloid fibers, we used Scanning Electron Microscopy (SEM) and Transmission Electron Microscopy (TEM) to compare the dsCsgA-Mfp3-A fusion strains and the wild-type strains (Figures 1B and S1). In both dsCsgA-Mfp3-A-producing strains, SEM revealed more amyloid fibers compared to the wild type, showing greater biofilm production likely due to the adhesive properties of the Mfp3-A functionalization (Figures 1B and S1). In fact, the monospecies biofilm of the fusion protein expressed with P­(dsCsgA) showed a complete curli extracellular fiber network. We did observe some extracellular fibers in the wild-type strain, but this observation is consistent with the natural basal production of amyloid fibers in .^29^ These observations were consistent with the TEM images, which also showed more pronounced and frequent fiber formation emanating from the plasma membrane in both dsCsgA-Mfp3-A strains compared to the wild type (Figure 1B,C and S1). Again, the fusion expressed with P­(dsCsgA) showed the most frequent fiber formation, with approximately 80% of the bacteria displaying this fibrous network (Figures 1Cand S1).

Next, we explored if these fiber networks could increase initial attachment and form more biofilm. Indeed, we observed an increase in bacterial attachment and biofilm formation in most variants carrying the dsCsgA-Mfp3 fusion using crystal violet staining on polystyrene plates. Both Mfp3-A and Mfp3-SP significantly increased biomass compared to the control, regardless of the promoter used. In contrast, the Mfp3-S variant showed increased adherent growth when expressed with the *aphII* promoter, and this effect was lower compared to the other variants (Figure 2A). Subsequently, centrifugal force was applied to the grown biofilms to assess the biofilm integrity of our engineered strains. The biofilm loss in the P­(dsCsgA)-dsCsgA-Mfp3-A variant was found to be lower than that in the strain containing the empty plasmid (Figure S2).

**Figure 2:**
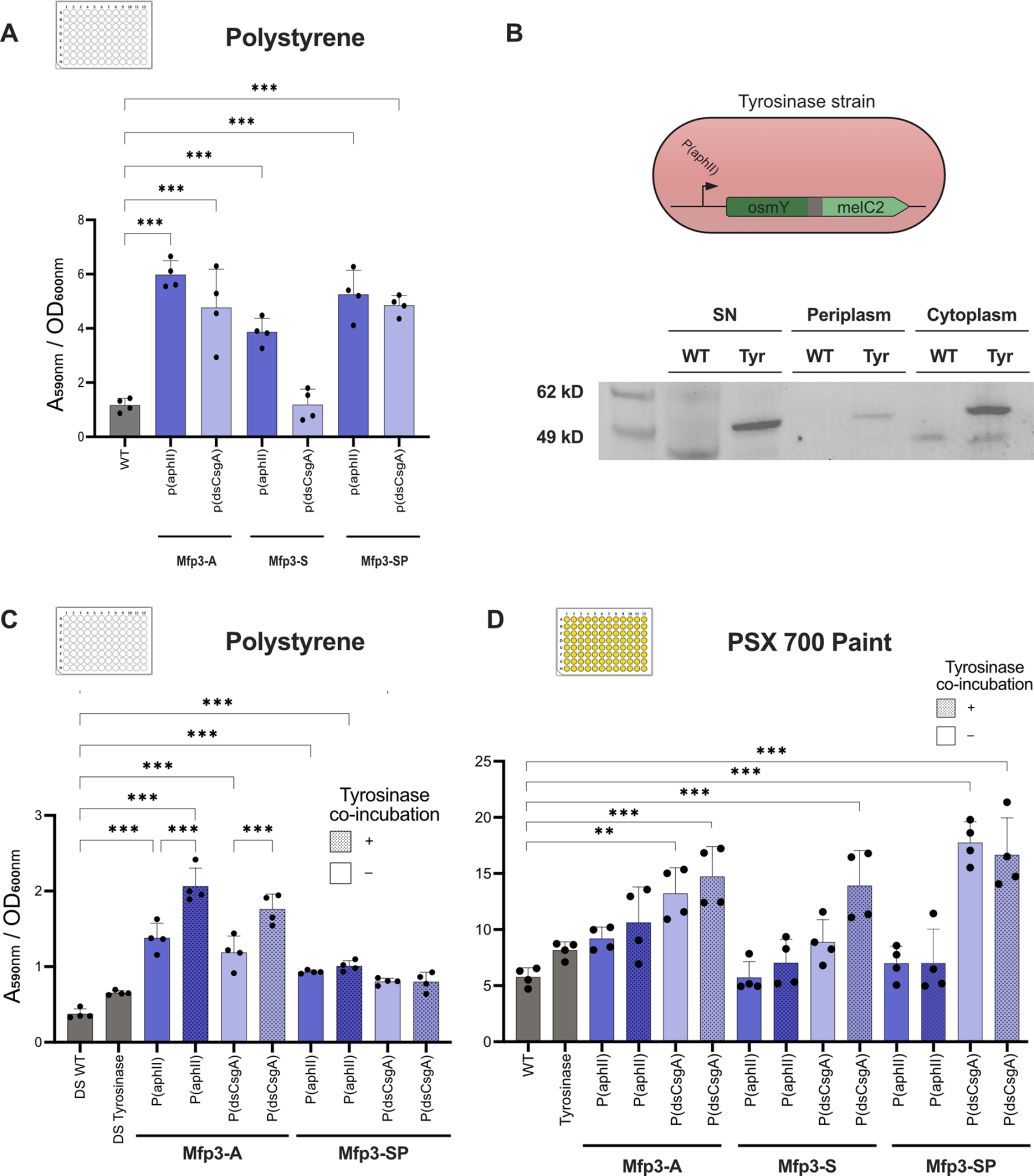
Enhanced biofilm formation in variants expressing dsCsgA and Mfp3 genes. (A) Biofilm formation values measured with crystal violet staining of the dsCsgA-Mfp3 variants compared to the wild-type on polystyrene plates. (B) Schematic representation of the tyrosinase construct, along with western blot analysis showing the OsmY-melC2 fusion protein (53.9 kDa). (C) Biofilm formation of the dsCsgA-Mfp3-A and dsCgsA-Mfp3-SP variants, with or without coincubation with tyrosinase producing strain on polystyrene plates. (D) Biofilm formation of the dsCsgA-Mfp3 strains, with or without coincubation with tyrosinase producing strain, on plates coated with PSX 700 paint. Data are presented as mean ± SD (*p ≤ 0.05, **p ≤ 0.01, ***p ≤ 0.001).

Tyrosinase postmodification of dsCsgA-Mfp3s fibrils significantly increased adhesion in .^23^ Therefore, we explored the combination of the tyrosinase enzyme from *sp*. with the dsCsgA-Mfp3 variants to potentially enhance microbial attachment and promote biofilm formation. We engineered a strain that harbors two distinct genes to ensure tyrosinase activity: *melC2*, which encodes the tyrosinase enzyme responsible for the modification, and *osmY*, a periplasmic protein that facilitates protein secretion. This strain produces an OsmY-melC2 fusion protein (Figure 2B). The expression of *melC2*, the key gene encoding the enzyme of interest, was confirmed via RT-qPCR (Figure S3), and we also confirmed via western blot that the fusion OsmY-melC2 protein was properly produced and secreted extracellularly (Figures 2B and S4).

Then, we evaluated whether this OsmY-melC2 producing strain would increase biofilm formation of the dsCsga-Mfp3 producing strains. We coincubated the tyrosinase-producing strain with the dsCsgA-Mfp3 variants at a ratio of 1:10 (Figure S5). Notably, harboring the dsCsgA-Mfp3-A variant exhibited increased biofilm formation, regardless of the promoter used, when coincubated with the tyrosinase producing strain on polystyrene plates. However, no increase in biomass was observed for the dsCsgA-Mfp3-SP variant (Figure 2C).

Once we validated our ability to enhance biofilm establishment on laboratory materials, we investigated whether the robust biofilm formation observed in our marine bacteria-based ELMs would also occur on commercial coatings currently used for submerged surfaces. Among these, PPG PSX 700 is a coating with excellent adhesion, corrosion, and chemical resistance, making it a suitable surface for deploying our ELMs.^30^ Therefore, we assessed the ability of our engineered strains to attach and grow on this paint, both with and without the addition of our tyrosinase-producing strain. Crystal violet staining showed that P­(dsCsgA)-dsCsgA-Mfp3-A and P­(dsCsgA)-dsCsgA-Mfp3-SP strains exhibited a significant increase in biofilm formation compared to that of wild type (Figure 2D). Additionally, the inclusion of the tyrosinase enzyme further enhanced biofilm formation in all P­(dsCsgA) variants. Although the increase was significant when compared to the wild type, no significant differences were observed within each variant, as shown in the results for polystyrene plates. This finding could be explained by the antifouling properties of the PSX 700 paint.^31^

### Domestication of Natural Heat Shock Response for Temperature Sensing

One of the main advantages of using living systems is the possibility of engineering dynamic responses to environmental stimuli and generating adaptive materials. After increasing the surface colonization of different strains, we explored how to measure key environmental cues to monitor biofilm state or to even regulate adhesion or other properties dynamically. We focused on characterizing transcriptional sensors for that are able to detect external signals. To simplify and quickly adapt biosensors in a non-model marine bacterium, we decided to domesticate endogenous regulatory systems for this detection. To uncover such endogenous systems already present in the genome, we exposed this bacterium to a temperature shock (42 °C) for 15 min to find changes in gene expression. We found 40 differentially expressed genes (LogFC > 5, FDR < 0.05) with 27% of unknown function proteins among them. Interestingly, around 17% of these genes were related to putative transposase family proteins. We also found common heat shock protein homologues to be upregulated, including the ATP-dependent chaperone ClpB (Dshi_0617), the chaperonin GroEL (Dshi_2919), the cochaperone GroES (Dshi_2920), the chaperone DnaK (Dshi_3571), the nucleotide exchange factor GrpE (Dshi_3465), and the heat inducible transcriptional repressor HrcA (Dshi_3464) (Figure 3A and File S1). One particularly interesting and highly upregulated locus comprehends Dshi_0075, a short hypothetical protein with 81 amino acids length next to another predicted hypothetical protein with 52 amino acids length. These two pseudogenes were both upregulated with fold changes of 8 and 9, respectively, and apparently belong to the same transcript (Figure 3B).

**Figure 3:**
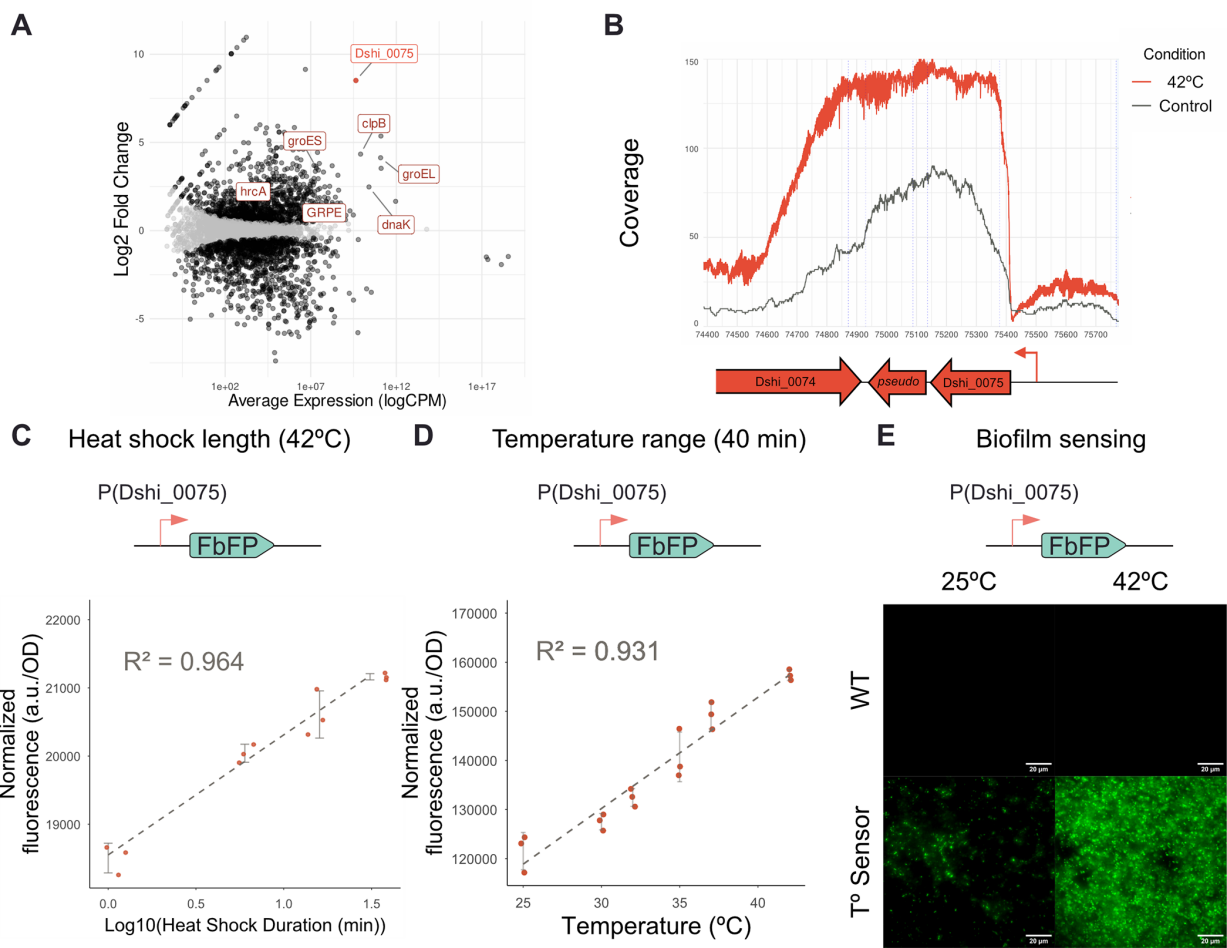
Natural response adaptation for temperature sensing. (A) MA plot of significantly (FDR < 0.05) upregulated genes. Genes highlighted correspond to canonical homologues related with the heat shock stress response. Dshi_0075 is highlighted as the selected gene. (B) RNA coverage at the Dshi_0075 genomic locus. The red line represents the average of the samples treated with 42 °C for 15 min whereas the gray line is the average control coverage. (C) FbFP fluorescent signal of the temperature biosensor strain after different times of heat sock treatment (5, 15, 30, 42 min) at different temperatures (32, 37, 42 °C). The diagram at the top depicts the genetic design of the temperature sensor architecture. (D) FbFP fluorescence of the temperature biosensor strain after 40 min of exposure to different temperatures. The diagram at the top depicts the genetic design of the temperature sensor architecture. (E) Representative fluorescent TIRF images of a biofilm formed by the wild-type (WT) and the temperature sensor strains (T° Sensor), exposed to either room temperature (25 °C) or heat shock (42 °C).

To create a temperature biosensor in , we selected 200 bp upstream of the Dshi_0075 start codon and introduced them in the replicative plasmid pBBR1MCS to control the expression of a Flavin-based Fluorescent Protein (FbFP).^32^ Then, we exposed the created strains to a range of temperatures (32, 37, and 42 °C) for different incubation times (5, 15, 30, and 45 min) and measured their fluorescence (Figure 3C). We observed an increase in FbFP fluorescence in for the three temperatures tested with just 5 min of incubation time, demonstrating the ability of Dshi_0075 promoter to be regulated by temperature (Figure 3C). In addition, the FbFP fluorescence increased linearly with fluorescence at 37 and 42 °C. However, we did not observe this linear behavior at 32 °C, where longer incubation times did not increase the response compared to the 5 min incubation (Figure 3C). This observation suggests that longer incubation times at 37 or 42 °C might also eventually plateau. Moreover, given that this endogenous system had not been characterized yet, we also explored if different temperatures could also induce the system. For this reason, we evaluated a range of temperatures from 25 to 42 °C for fluorescent activation of the temperature-sensing strain with an incubation time of 40 min. We observed a linear increase of fluorescence with temperature starting at 32 °C (Figure 3D).

Finally, we decided to test whether this temperature-sensing strain is active within a biofilm and be eventually integrated into a biofilm-forming and temperature-responsive ELM. To achieve this, we imaged biofilms of the wild-type and temperature-sensing strains when exposed to either 25 or 42 °C for 2 h. Indeed, we observed the temperature-sensing strain to have increased fluorescence across the biofilm (Figure 3E). We also observed an increased biofilm fluorescence in 96-well plates when they were exposed to 42 °C shock for 1 h, and all planktonic bacteria were washed (Figure S6).

### Engineered Detects Oxygen Availability

Oxygen gradients within biofilms modulate bacterial metabolism and, eventually, adhesion.^33^ For this reason, we explored how to measure available oxygen for our engineered strains, not only for ocean monitoring but also to ultimately regulate biofilm structure using oxygen.

Although previously was believed to be strictly aerobic, this bacterium is able to grow in anaerobic conditions using nitrate as the terminal electron acceptor.^21^ The aerobic to anaerobic transition is known to be regulated by the FnrL transcription factor (TF). FnrL senses oxygen through the oxidation of an Fe–S cluster and can act as a repressor or an activator depending on the binding location.^34^ Following the same approach as with the temperature biosensor, we decided to adapt the natural transcription factor in to ensure the functionality of the system. In this case, we selected the promoter from *hemN2* (Dshi_0659), previously reported to be upregulated in anaerobic conditions in .^34^ This gene is also located upstream of *fnrL* (Dshi_0660). Both genes are divergent and share the intergenic region, a common feature in bacterial operons where the regulator is usually opposite to the genes it directly regulates.^35^ For this reason, we designed a plasmid (pOxy-A) harboring the *hemN2* promoter driving the expression of FbFP. In addition, we also created another plasmid (pOxy-B) with the same *hemN2*-FbFP transcriptional unit and also expressed the *fnrL* gene using the constitutive promoter *aphII,* shown previously to work in^21^ (Figure 4A). We also chose to use FbFP to avoid differences in fluorescence signal due to oxygen maturation, as common fluorescent reporters require oxygen to mature. Both plasmids showed a significant increase in FbFP after growth for 24 h in anaerobic conditions compared to an aerobic culture. Surprisingly, the constitutive expression of FnrL yielded a smaller fluorescence increase than the *hemN2* promoter alone (Figure 4B).

**Figure 4:**
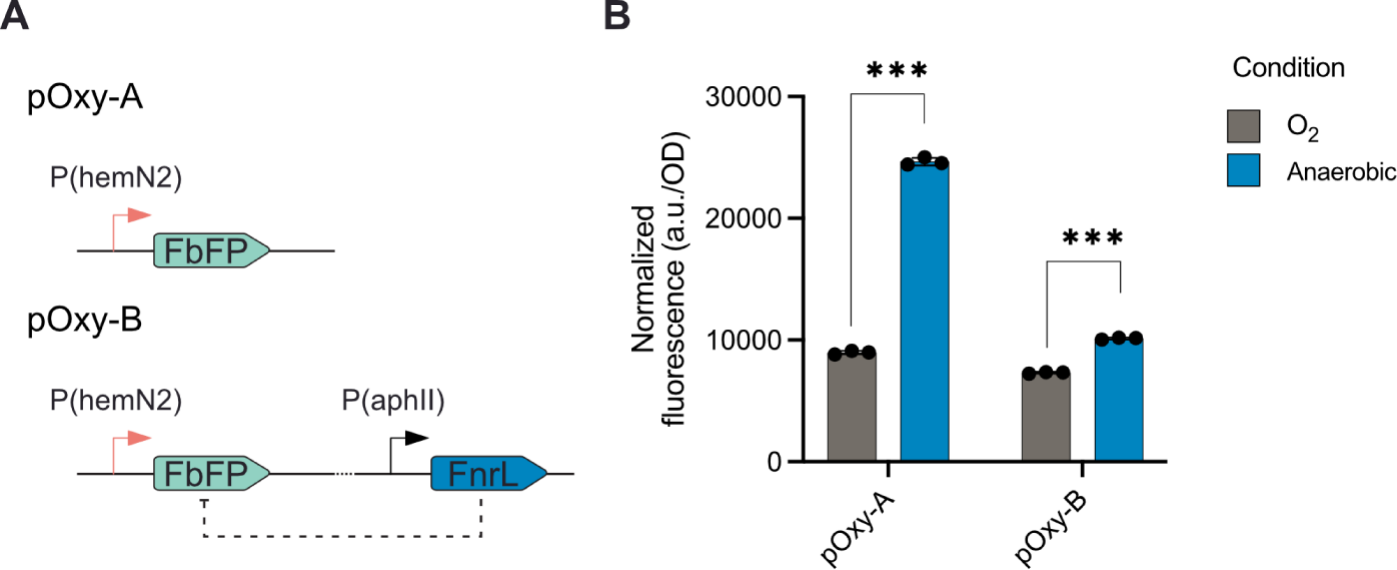
FnrL regulated promoter adaptation for oxygen sensing. (A) Schematics of the two alternative plasmid designs for oxygen sensing pOxy-A and pOxy-B. (B) FbFP fluorescent values of the two different oxygen biosensors for absence and presence of oxygen (*** *p* < 0.001).

### Stable Recording of Expression for Biosensor Monitoring

One of the main challenges for bacterial whole cell biosensor deployment in the environment is the transient nature of gene expression. Whole cell sensors might offer more precise information on how temperature and oxygen are actually bioavailable to the microorganisms on the ship’s surface. Engineering the ocean microbiome for biosensing strategies requires continuous monitoring of the bacterial expression to understand the dynamics of the measured signals.

To solve this issue, we focused on implementing a synthetic memory system based on the RT-Cas1-Cas2 complex from .^27^ This system can store spacers from RNA in a specific CRISPR array, transforming a temporal transcriptional signal into a stable DNA archive that can be retrieved at convenience (Figure 5A). To implement such a system for the development of sentinel cells, we expressed the RT-Cas1 and Cas2 proteins under the control of constitutive promoter *aphII* and included them in the plasmid together with the CRISPR array where the RNA spacers are acquired. Using the SENECA selective amplification method, we compared the spacers acquired for two 10 h bacterial cultures, one kept at normal 30 °C and the other grown at 42 °C. We observed the spacers acquired to have the expected length distribution and GC content (Figure 5B, C). Based on the spacers aligning to the genome and the plasmid, we calculated the spacer counts per gene and were able to distinguish, using a Principal Component Analysis (PCA), those samples treated with heat shock from the control (Figure S7A). Samples were also clustered by treatment using an unsupervised hierarchical clustering of the genome aligned counts (Figure S7B).

**Figure 5:**
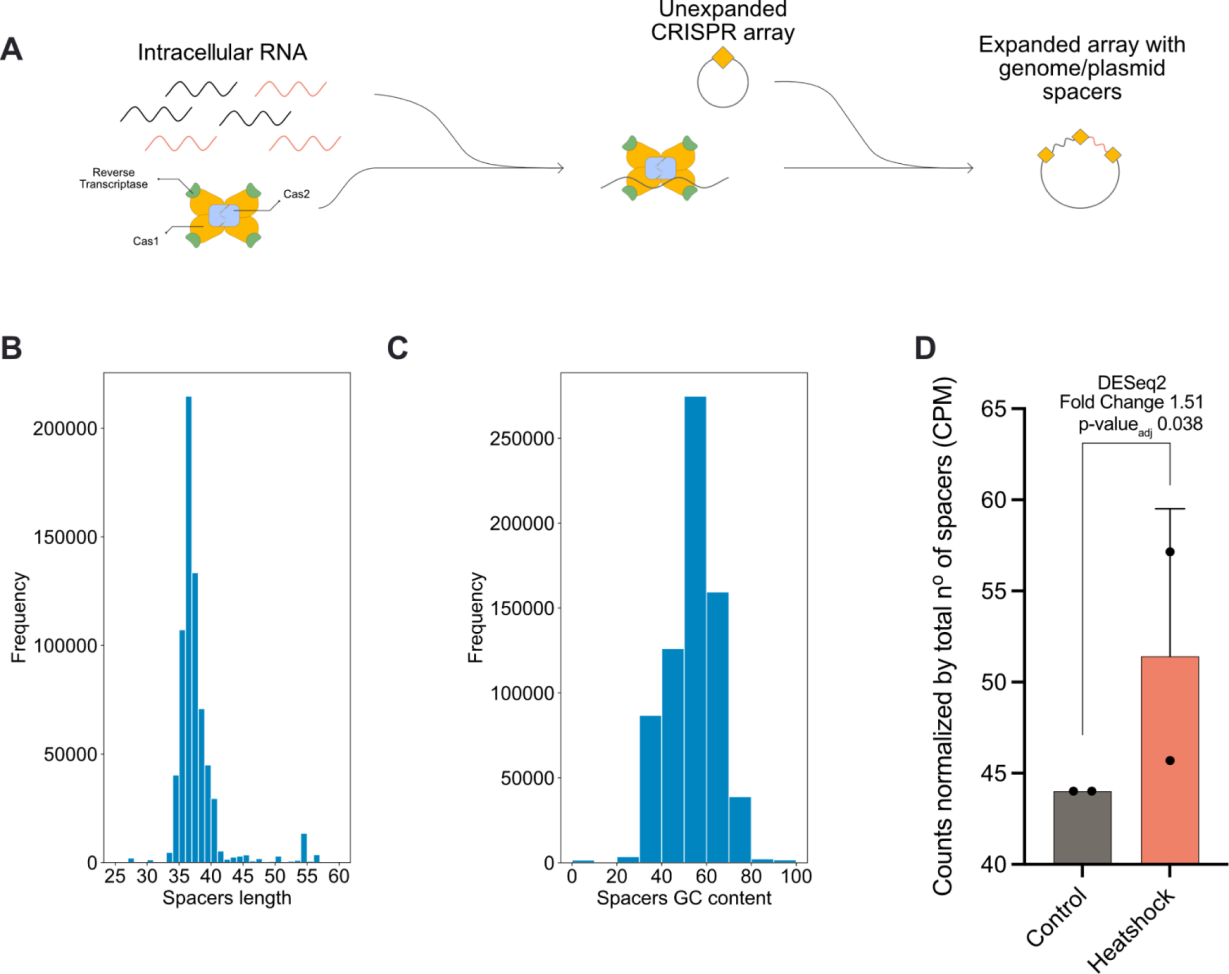
Sentinel stores transcriptional information in DNA using Record-Seq. (A) Schematics of the Record-seq recording technology. (B) Length distribution of the acquired spacers. (C) GC content distribution of the acquired spacers. (D) Relative spacer counts of FbFP aligning spacers between the 42 °C treated and the control sample, DESeq2 computed fold-change and adjusted *p* value are reported at the top.

We also explored whether unbiased RNA recording could be directed to arbitrarily record one of our biosensors. To test this hypothesis, we included our temperature biosensor and the recording machinery in the same plasmid. We again compared the spacers recorded in a 42 °C treated sample with a control sample. Indeed, we observed an increase in the recorded spacers corresponding to the FbFP. Furthermore, after testing for differential expression with DESeq2, FbFP showed significant upregulation in treated samples when compared against the control ones (Figure 5D). These results demonstrate that we are able to combine the programmed dynamics of our biosensors with stable signal recording.

## Discussion

In this study, we demonstrated the suitability of as a platform for developing marine ELMs. As a proof of concept, we engineered this bacterium to have increased bacterial attachment and biofilm formation, detect environmental signals, and record its transient transcription into the genome.

To increase the initial attachment and biofilm formation of , we engineered different strains that combine two independent natural adhesion systems: amyloid-based and DOPA-based adhesives. Amyloid structures, found in certain bacterial biofilms and fungal hydrophobins, contribute to biofilm robustness, while DOPA-based adhesives, like those in mussel foot proteins, enable attachment to submerged surfaces. Previous work showed that CsgA amyloid fibrils and Mfp3 mussel proteins can act as molecular glues in .^23^ However, is an intestinal bacterium not suitable for marine environments. We engineered the endogenous CsgA fibrils of and introduced the Mfp3 mussel protein domain to increase adhesion in a marine-relevant microorganism. SEM and TEM microscopy showed the production of an extensive fiber network by our engineered strains compared to that of wild type.

Our results suggest that the engineered strains exhibit enhanced surface-associated growth, potentially enabling protective biofilm formation on submerged surfaces. Both Mfp3-A and Mfp3-SP variants showed increased biofilm-forming capabilities compared to the wild type, regardless of the promoter used. These candidates were selected for coexpression with the tyrosinase enzyme from sp. which we confirmed to be released into the extracellular environment. When coexpressed with Mfp3, this enzyme enhances the adhesive properties of Mfp3 through the conversion of tyrosine residues to 3,4-dihydroxyphenylalanine (DOPA).^36^ After coincubation, crystal violet staining revealed increased biofilm development in P­(aphII)-dsCsgA-Mfp3-A and P­(dsCsgA)-dsCsgA-Mfp3-A variants. Interestingly, although the percentage of tyrosine amino acids is approximately 22% in all Mfp3 proteins, the tyrosinase enzyme appears to have an increased propensity to convert tyrosine residues of Mfp3-A to DOPA, thereby maximizing the number of adhesive bonds formed on our target surface. Additionally, lysine residues near DOPA in mussel foot proteins help displace salt and water molecules, improving DOPA binding.^37^ Mfp3-A contains twice as many lysine residues as Mfp3-SP, which likely explains the improved bacterial attachment, ultimately promoting more substantial biofilm formation.

We also assessed the growth of ELMs on the PPG PSX 700 coating paint, which provides excellent adhesion, toughness, corrosion resistance, chemical resistance, and suitability for immersion services, as well as low environmental impact. However, these coatings are unable to prevent biofouling in the long term under certain environmental conditions. Our goal is to leverage the advantages of ELMs to counteract these PPG PSX 700 limitations. Crystal violet results showed increased biofilm formation in P­(dsCsgA)-dsCsgA-Mfp3-A and P­(dsCsgA)-dsCsgA-Mfp3-SP strains. Coincubation with tyrosinase resulted in increased biofilm formation compared with the wild type, although the increase was not statistically significant when compared to the Mfp3 strains alone. This is coherent with the antifouling effect of the PSX 700 paint, which may impair DOPA residue adhesion due to the polysiloxane nature of the coating.^31^

Our results show that the strain P­(dsCsgA)-dsCsgA-Mfp3-A forms an extensive fiber network in SEM and TEM images, promotes consistent biofilm formation on two hydrophobic materials, and preserves biofilm integrity after centrifugal force application. Additionally, coincubation with tyrosinase producing strain significantly increased biofilm biomass on polystyrene and showed an increasing trend on PSX700 coating paint.

These results represent a proof of concept for engineering the non-model organism , enhancing its early colonization ability to form biofilms with increased biomass. By promoting colonization resistance through competitive exclusion, the engineered strains could serve as an environmentally friendly strategy to help protect submerged surfaces against unwanted microbial colonization and, ultimately, biofouling.^38–40^ To our knowledge, this is the first time that the combination of CsgA and Mfp3 proteins has been introduced into an early marine surface colonizer such as . Furthermore, we have assessed DOPA binding through hydrophobic interactions not only in polystyrene but also on commercially available coatings, advancing our application toward realistic deployment. Nonetheless, more research is required to elucidate the mechanisms behind this phenomenon in . Additionally, further studies are needed to examine other surface interactions as well as key microbial processes associated with surface colonization, such as community sensing and signaling, intraspecific and interspecific communication and interaction, and the balance between cooperation and competition.

One key advantage of using living bacteria for coating applications is their ability to be genetically engineered for dynamic environmental sensing and control. In this study, we focused on the engineering of transcriptional sensors in to later equip our biofilms with more capabilities. However, the lack of portability of transcription factors across species makes it a difficult task to establish new biosensors in non-model bacteria.^41^ For this reason, we focused on the natural genetic resources within to establish new oxygen and temperature sensors for this marine bacterium. We demonstrated that the adaptation of the natural transcriptional response represents a rapid strategy for quickly validating biosensors, as we simply adapted the regulatory sequences from the differentially expressed genes or took already available data^34^ to define the inducible promoter sequences. Additionally, these sensors represent a starting point to generate sensing and acting biofilms in marine environments, as they can be coupled with effector proteins to increase the biofilm adaptability or to produce antifouling metabolites under specific conditions. In fact, high temperature adaptation increases biofilm formation in ,^42^ thus inducing biofilm formation using synthetic gene circuits could programmatically enhance the resistance of engineered in a rational manner.

The transcriptional characterization of the temperature shock response of found several homologues commonly associated with temperature stress response in other bacteria including effectors such as chaperone DnaK or regulators such as HrcA. However, we could not find the characteristic CIRCE DNA binding motifs from the HrcA regulator within the Dshi_0075 promoter.^43^ Furthermore, Dshi_0075 has no direct predicted function based on homology with other known proteins. The analysis of the amino acid sequence for motifs reveals a predicted signal peptide for secretion and a putative EF-hand domain involved in calcium binding. Several prokaryotic calcium binding proteins have been associated with heat shock response regulation^44^ , indicating that this protein might be involved with Ca^2+^ in heat shock response regulation. Despite not being able to predict the gene function of Dshi_0075, we were able to readapt its regulatory sequences for temperature detection, even within an actual biofilm. However, further information on which transcription factor regulates this promoter would enable us to further fine-tune the temperature sensor response.

In the case of our oxygen sensor, the ability to pinpoint the genetic changes to a specific FnrL regulator further allowed us to fine-tune the sensor response in the absence of oxygen. We successfully adapted the already characterized transcriptional regulation for anaerobic conditions described in^34^ with the FnrL regulator to create an anaerobic activation sensor. Interestingly, the transcription factor constitutive expression led to a reduction in the fold-change activation of the system. An increased concentration of transcriptional repressors usually reduces the basal signal but induces general activation once the signal is present. However, an excess of transcriptional repressor concentration can also reduce the overall response, as the high concentration can compensate for the reduced binding affinity of the transcription factor. For this reason, further experiments are required to elucidate the role of FnrL in regulating HemN2.

However, further characterization of these sensors might be required to ensure that their activation is specific to the signals we describe and are not activated in other general stress responses. Our oxygen sensor has homologous genes related to oxygen metabolism regulation in other bacteria. In contrast, the temperature sensitive promoter we developed comes from a hypothetical protein that might participate in other responses, although its upregulation correlates with well-known heat shock regulatory genes.

Marine bacterial adapts their gene expression to accommodate the environment even when perturbations appear, such as pollution or temperature. Monitoring this gene expression enables the retrieval of the complex effect of these alterations in biological ecosystems, providing more information compared to other types of sensing. We demonstrated the portability of the Record-seq technology in to record stable information from transient RNA expression. We also directed the untargeted expression recording of this system toward the specific recording of target RNA signals by coupling it with our transcriptional temperature sensor, demonstrating the possibility of designed multiplex biosensor recording to store longitudinal information in real life situations. Later retrieval of these sequences enables us to understand the expression history of in different marine conditions, with the potential to noninvasive monitoring of gene expression even as an adhered biofilm in the ship surface.

In conclusion, here we proposed engineered for applications in the marine environment. We have not only improved biofilm-forming capabilities but also found new biosensors that, coupled with Record-seq technology, could record specific signals. These technologies will be useful for the development of smart biofilms that could sense and permanently record changes in the environment. Although the application of ELMs in real environments requires further testing, the strains described in this publication represent a first step in the use of as an example of the potential of microbiome engineering for marine applications.

## Methods

### Strains and General Growth Conditions

 strain DFL-12 was obtained from the German Collection of Microorganisms and Cell Cultures (DSMZ)­(DSM 16493). For all experiments, single colonies were cultured in Marine Broth (MB) (no. 279110, Difco) for 48 or 72 h at 30 °C with shaking at 200 rpm. Exponentially growing cultures were prepared by reinoculating a 72 h grown culture at 10% in fresh MB media and grown for 24 h at 30 °C with 200 rpm shaking.

Plasmids were transformed into chemically competent DH5α (MB00402, NZYtech). This strain was grown at 37 °C either in LB agar plates or shaking LB liquid cultures with the appropriate antibiotic (50 μg mL^–1^ Kanamycin or 25 μg mL^–1^ Chloramphenicol).

For conjugation experiments, we used the DSMZ strain ST18 (DSM 22074). This strain is a hemA mutant of the λ-pir strain of S17 capable of performing conjugation but is auxotrophic for aminolevulinic acid (ALA), the central precursor of tetrapyrrole, and requires its supplementation for growth. This strain was grown on LB or hMB agar plates, in liquid cultures with shaking, and with the appropriate antibiotic according to the experiment (50 μg mL^–1^ Ampicillin, 50 μg mL^–1^ Kanamycin, 50 μg mL^–1^ Spectinomycin, or 25 μg mL^–1^ Chloramphenicol) and auxotrophy supplementation in the presence of 25 μg mL^–1^ of aminolevulinic acid (ALA) (A7793-500MG, Sigma-Aldrich).

### Plasmids and Cloning

All plasmids used in this study were based on the pBBR1MCS replicative vector.^21^ PCR fragments were usually amplified with either KAPA HiFi (KK2601, Roche) or Phanta Max (#001, Vazyme) and purified using QIAquick PCR purification or gel extraction kits (#28104/#28704, Qiagen). When necessary, plasmids were digested with the corresponding restriction enzymes (NEB) and assembled by using a custom Gibson enzyme mix (Center for Genomic Regulation CRG, Barcelona). The CsgA (Dshi_0598) sequence was obtained from the DFL-12 reference genome (RefSeq: GCF_000018145.1). The *mfp3* sequences were obtained from previous publications: Mfp3-A,^23^ Mfp3-S,^28^ and Mfp3-SP.^5^ The *melC2 and osmY* genes were obtained from a previous publication.^28^

### ST18 Cell Preparation and Electroporation

ST18 chemically competent cells were prepared as previously described.^45^ Briefly, an exponentially growing bacterial culture in LB supplemented with ALA (50 μg mL^–1^) was harvested at 0.4–0.6 OD_600 nm_ by centrifuging at 4500 rcf and 4 °C for 10 min. Afterward, three subsequent washes with Cacl_2_ solution (Cacl_2_ 60 mM, Tris-HCL 10 mM, glycerol 15%) were performed at 4500 rcf 4 °C for 10 min each. Subsequently, cells were kept on ice for 30 min. Finally, cells were centrifuged, resuspended in ultrapure water with 10% glycerol, aliquoted, and stored at −80 °C for later use.

For electroporation, 25 μl of ST18 electrocompetent cells were mixed with the desired plasmid and electroported with a gene pulser electroporator (Biorad) at 25 μF, 200 Ω, and 1.8 kV. After electroporation, cells were resuspended in 300 μL of SOC media and incubated for recovery for 1 h . Cells were then plated on LB supplemented with 25 μg mL^–1^ chloramphenicol and 50 μg mL^–1^ ALA.

###  Conjugation

The conjugation of plasmids from a donor ST18 to a recipient was performed as previously described with slight adjustments.^21^ Briefly, donor ST18 cells were grown overnight at 37 °C in LB medium supplemented with 50 μg mL^–1^ ALA (A7793-500MG, Sigma-Aldrich). The next day, the culture was diluted five times (1:4) with ALA supplemented LB medium and incubated at 37 °C for 3-4 h to allow the bacteria to reach the exponential phase. In parallel, cultures were grown for 72 h in MB, and 24 h prior to the conjugation assay, and they were diluted in half (1:1) with MB medium to obtain exponential phase bacteria. On the day of the conjugation, both cultures ST18 and were diluted to an OD_600 nm_ equal to 1 and mixed in a donor:recipient ratio of 10:1 to a 2 mL final volume. This mixture was centrifuged for 2 min at 800 rcf, and the pellet obtained was resuspended in 150 μL of MB. The entire volume was deposited dropwise in the center of an hMB plate supplemented with 50 μg mL^–1^ ALA and incubated with the plate facing upward at 30 °C for a period of 48 h. To obtain isolated colonies, the grown culture was resuspended in 200 μL of MB and plated on MB plates supplemented with 6.25 μg mL^–1^ chloramphenicol and incubated at 30 °C for 1 week to obtain single conjugated colonies.

### Biofilm Formation Assays

Bacterial biofilm formation was quantified according to the crystal violet as previously reported with some modifications.^46^ Bacterial strains were grown for 48 h with shaking at 30 °C and diluted to an OD_600 nm_ of 0.1. 200 μL of each culture, which were transferred in triplicate to 96-well plates (003596, Corning) and cultured for 7 days at 30 °C without shaking in a humid chamber. After one week, the OD_600 nm_ was measured using the M Nano Infinite 200 Pro plate reader (Tecan). The planktonic cultures were aspirated, and each well was washed three times with 150 μL of PBS. 160 μL of 0.5% filtered crystal violet was added, shaken at 100 rpm for 20 min at room temperature, and covered with aluminum foil. After incubation, the excess crystal violet was aspirated, and the wells were washed 3 times with PBS. To measure biofilm biomass, 200 μL of 100% ethanol was added to each well, and the plate was shaken for 20 min at room temperature, protected from environmental light. Finally, the A_590 nm_ was measured using an M Nano Infinite 200 Pro plate reader (Tecan), and the A_590 nm_/OD_600 nm._ ratios were calculated to determine biofilm biomass.

To evaluate the effect of the tyrosinase-producing strain, the P­(aphII)-dsCsgA-Mfp3-SP variant was grown in the presence of the tyrosinase strain at different ratios, and biofilm formation was determined by using the previously described crystal violet assay. Strains were grown to the exponential phase, diluted to an equal OD_600 nm_ of 1, and cultured according to the different tyrosinase/P­(aphII)-dsCsga-Mfp3-SP ratios. The cultures were then incubated for 1 week at 30 °C without shaking in a humid chamber. The optimal ratio was selected for subsequent assays (Figure S2).

For the 96-well plates (003596, Corning) coated with PSX700, the protocol followed was the same as the one described above with some modifications. To measure the OD_600 nm_ and A_590 nm_, 100 μL of bacterial culture was transferred into a polystyrene 96-well plate.

### Immune Detection of Tyrosinase

Periplasmic and cytoplasmic contents were prepared as follows: 1.5 mL of bacterial cells were pelleted (8000 rcf, 2 min) and resuspended in 200 μL of shock buffer (100 mM Tris–HCl, pH 7.4, 20% sucrose (w/v), and 10 mM EDTA). The suspension was incubated on ice for 5 min. After centrifugation (8000 rcf, 2 min), the pellet was rapidly resuspended in 200 μL of water with vigorous shaking. The suspension was incubated on ice for an additional 5 min and centrifuged at 16,000 rcf for 2 min. The pelleted cells were resuspended in 200 μL of water and lysed using Precellys 0.1 mm silica beads (432-3754, VWR) in a Precellys Cryolys Evolution instrument (Bertin Instruments). The soluble fraction (periplasmic content) was collected by centrifugation (16,000 rcf, 2 min), while the pellet was washed and resuspended in 200 μL of distilled water, representing the cytoplasmic fraction.

The SN that was kept on ice was processed via trichloroacetic acid (TCA) precipitation. Samples were mixed with 10% TCA and incubated on ice for 30 min. The precipitate was pelleted by centrifugation (∼10,000 rcf, 10 min, 4 °C), washed twice with 500 μL of cold (−20 °C) acetone, air-dried, and resuspended in PBS.

Periplasmic, cytoplasmic content, and SN were analyzed using SDS-PAGE and His-tag immunodetection. Protein concentration was normalized to 10 μg per sample. Briefly, a NuPAGE 4–12% gel (NP0322BOX, Invitrogen) was run at 120 V for 1 h 30 min to 1 h 45 min, and proteins were transferred onto a PVDF membrane (IPVH00010, Immobilon-P Transfer Membrane, Merck Millipore) using a wet blotting apparatus running at 20 V for 1 h. Membranes were blocked (1 h at room temperature or overnight at 4 °C) with 4% non-fat milk in TBST (Tris-buffered saline (1706435, Bio-Rad) containing 0.05% Tween 80) and incubated (1 h at room temperature or overnight at 4 °C) with mouse anti-His antibody (MCA1396GA, Bio-Rad) diluted 1:800 in TBST-4% milk. Following three 10 min washes in TBST, membranes were incubated for 1 h in horseradish peroxidase-coupled antimouse antibody (sc516102, SantaCruz) diluted 1:1000 in TBST-4% milk and washed again three times 10 min each in TBST. Membranes were developed with the Pierce ECL western Blotting Substrate (32106, Thermo Fisher Scientific) and recorded using a ChemiDoc MP Imaging System (Bio-Rad).

### Scanning Electron Microscopy (SEM)

For scanning microscope analysis, samples were deposited on poly l-lysine coverslips and fixed in a solution consisting of 2.5% glutaraldehyde in 0.1 M phosphate buffer (pH 7.4), postfixed in osmium tetroxide (1%) in the same phosphate buffer, dehydrated in graded alcohol, and processed for critical point drying using Emitech K850. Samples were covered with a carbon thin film in order to improve their electrical conductivity. The samples were observed with a Jeol JSM-7001F instrument (Jeol, Japan) operated at 15 kV in the TEM-SEM Electron Microscopy Unit of the Scientific and Technological Centers (CCiTUB), Universitat de Barcelona.

### Transmission Electron Microscopy (TEM)

The sample pellet was fixed in 2% paraformaldehyde, 2.5% glutaraldehyde and 0.1 M phosphate buffer and incubated at 4 °C for 30 min in the shaker. After centrifugation at 2500 rpm for 5 min, the samples were washed at 4 °C for 10 min in the fixation buffer and washed four times for 10 min with PB 0.1 M, pH 7.4 at 4 °C. Then, a solution of 1% osmium tetroxide, 0.8% potassium ferrocyanide, and 0.1 M PB pH 7.4 was added to the sample and incubated for 1.5 h at 4 °C in the dark and washed 4 times for 10 min with double-distilled water at 4 °C to eliminate excess osmium. After the sample was dehydrated with increasing concentrations of acetone, infiltration into the Spurr resin was performed followed by polymerization. Ultrathin 60 nm sections of the resin stub were cut using a Leica UC7 ultramicrotome and stained with aqueous uranyl acetate and Reynolds lead citrate before observation on a J1010 transmission electron microscope (JEOL) coupled with an Orius CCD camera (Gatan). Sections were imaged at 80 kV. TEM was performed at the TEM-SEM Electron Microscopy Unit of the Scientific and Technological Centers (CCiTUB), Universitat de Barcelona.

### Fluorescence Microscopy

Bacterial strains were grown for 48 h with shaking at 30 °C and diluted to an OD_600 nm_ of 0.8. 200 μL of each culture were transferred to μ-Slide 8-well ibiTreat plates (cat. #80826, ibidi) and cultured for 20 days at 30 °C without shaking in a humid chamber. To analyze the response of the temperature sensor within a biofilm after a heat shock, images were taken under two conditions: (1) control, where the culture equilibrated at room temperature for 1 h, and (2) heat shock, where the culture was exposed to 42 °C for 2 h. Images were captured immediately after the heat shock treatment. Images were acquired with total internal reflection fluorescence (TIRF) microscopy using the ECLIPSE Ti2-E inverted microscope (Nikon) with LED GFP at 100% power and a 200 ms exposure. The images were analyzed using ImageJ2 software (version 2.14.0/1.54f, open-source image processing software).

### Centrifugation Cell Adhesion Assay

Bacterial strains were grown for 48 h at 30 °C with shaking and diluted to an OD_600 nm_ of 0.1. 200 μL of each culture was transferred in triplicate to two 96-well plates (003596, Corning) and incubated for 7 days at 30 °C in a humidity chamber without shaking. OD_600 nm_ measurements were performed on both plates, and the planktonic cultures were aspirated. One plate served as a control, while the other was inverted, sealed with parafilm, and centrifuged at 500 rcf for 5 min to dislodge loosely attached cells. After centrifugation, both plates were washed three times with 150 μL of PBS. Adherent biofilms were stained with 160 μL of 0.5% filtered crystal violet, incubated for 20 min at room temperature with gentle shaking (100 rpm), and protected from light. Excess stain was aspirated, and wells were washed three times with PBS. To quantify adherence, 200 μL of 100% ethanol was added to each well to dissolve the crystal violet, and _A590 nm_ was measured using a Tecan M Nano Infinite 200 Pro plate reader. Biofilm adherence was calculated as A_590 nm_/OD_600 nm_. The ratio from the centrifuged plate was compared to the control plate to determine the percentage of bacterial cells lost after applying the dislodgement force.

### RNA Isolation

Exponentially growing cultures were exposed for 15 min to either a 42 °C heat shock or a 30 °C control temperature. Then, bacteria were harvested by centrifuging at 8000 rcf and 4 °C for 10 min and resuspended in RNA Protect Bacteria Reagent (76506, Qiagen). Then, bacteria were centrifuged again at 8000 rcf and 4 °C for 10 min, and the pellets were frozen in liquid nitrogen and stored at −80 °C for further processing. Total RNA was extracted using the miRNeasy kit (217004, Qiagen) according to the manufacturer’s instructions. Briefly, bacterial pellets were resuspended in 1 mL preheated 65 °C Qiazol (79306, Qiagen). Then, cells were lysed using Precellys 0.1 mm silica beads (432-3754, VWR) for 15 min in a Disruptor Genie (SI-D258, Scientific Industries). 200 μL of chloroform were added, and the samples were centrifuged at 12 000 rcf and 4 °C for 15 min. The upper aqueous phase was extracted, and 500 μL of fresh 80% ethanol were added. Then, the whole sample was transferred to the kit’s column and underwent subsequent centrifugation rounds at 8000 rcf for 15 s adding one round with 700 μL of Buffer RWT, two rounds with 500 μL of RPE Buffer, and finally eluting with 30 μL of RNase free water. Concentration was measured with a NanoDrop One Spectrophotometer (ND-ONE-W, ThermoFisher).

### RNA-Seq

Isolated RNA was analyzed for purity and integrity using Bioanalyzer (Agilent Technologies GmbH, Germany). Library construction and RNA sequencing were performed by Macrogen Inc. (Seoul, South Korea) using the Truseq Stranded Total RNA kit and sequenced using Illumina at 60 M pair-reads depth. RNA-seq analysis was performed using the nf-core RNA-seq pipeline v3.055,56 in Nextflow v20.12.0-edge.^47^ Raw paired-end reads were trimmed using Trim Galore v0.6.6 and aligned to the DFL-12 reference genome (GenBank CP000830) using STAR v2.6.1d^48^ and SAMtools v1.10.^49^ Quality control was performed using FastQC v0.11.9. Mapped reads were counted using mpileup from BCFtools in htslib v1.1. For temperature-sensitive promoters, differential gene expression analysis between three heat shock samples and three controls was performed on the normalized read counts using EdgeR.^50^ Genes with a log2 fold-change greater than 8 were selected for further manual inspection of the read coverage across the genetic locus. We extracted either 200 bp upstream of the start codon or the whole intergenic region for those genes whose upregulation was consistent all over their ORF.

### Plate Reader Assays

For temperature sensor experiments, exponentially growing temperature sensor strain cultures with 25 μg mL^–1^ chloramphenicol were diluted to 0.1 OD_600 nm_ and subjected to either a heat shock treatment for variable time or to different temperatures. Then, the cultures were distributed in a 96-well plate (003596, Corning) and left to grow 24 h at 30 °C in an M Nano Infinite 200 Pro plate reader (Tecan) measuring OD_600 nm_ and FbFP em/ex wavelength of 460/492 nm. To measure temperature sensor activity in biofilms, exponentially growing cultures were diluted to 0.5 OD_600 nm_ and grown at 30 °C in two 96-well plates (003596, Corning) for 12 days to allow biofilm formation. After this period, one plate was subjected to heat shock treatment for 1 h at 42 °C, and the other one was left at 25 °C. Then, OD_600 nm_ was measured using an M Nano Infinite 200 Pro plate reader (Tecan) to quantify planktonic bacteria. Both plates were washed with fresh marine broth medium to remove bacteria in suspension. Finally, we measured the FbFP em/ex wavelength of 460/492 nm fluorescence values in the remaining biofilm. For oxygen sensor experiments, the exponentially growing oxygen sensor candidates were grown to exponential phase, diluted to 0.1 OD_600 nm_, and grown for 24 h under aerobic or anaerobic conditions. Anaerobic conditions were generated using either the GasPak EZ anaerobe pouch system (no. BD260683, BD) or the AnaeroGen System (AN0025A, Thermo Scientific). After 24 h, all cultures were exposed to oxygen, distributed in a 96-well plate (003596, Corning), and measured in the plate reader under the same previously described conditions.

### Record-Seq

Exponentially growing cultures of with the recording plasmid or the recording plasmid together with the temperature sensor were exposed to either 42 or 30 °C for 8 h and harvested by centrifugation at 8000 rcf and 4 °C for 10 min. Bacterial pellets were lysed using Precellys 0.1 mm silica beads (432-3754, VWR) for 15 min in a Disruptor Genie (SI-D258, Scientific Industries) and then plasmid DNA was extracted using the NZYMiniprep kit (MB01008, NZYtech) according to the manufacturer’s instructions. Purified plasmids were selectively amplified for expanded Record-seq arrays (SENECA) as previously described.^51^ Briefly, the plasmids were digested with FaqI for an adapter ligation (85 cycles of 37 °C for 5 min and 20 °C for 5 min followed by 15 min at 55 °C) that was later amplified during a first PCR round (98 °C for 30 s; 25 cycles at 98 °C for 10 s, 57 °C for 30 s, and 72 °C for 20 s followed by 72 °C for 5 min). Then, PCR products were purified using AMPure XP beads (A63881, Beckman Coulter), and a subsequent 10 cycle PCR reaction for Illumina adapter ligation was performed (98 °C for 30 s, 10 cycles of 98 °C for 10 s, 65 °C for 30 s and 72 °C for 30 s, and 72 °C for 5 min). Prepared libraries were then loaded in a 2% EX E-gel (G401002, ThermoFisher) and gel extracted using the QIAquick gel extraction kit (28704, Quiagen). Concentration and size distribution of the libraries were validated using a TapeStation Screentape D1000 (Agilent Technologies). An equimolar pool of the libraries was prepared and quantified by qPCR with a Light Cycler (Roche). The final pool was denatured and diluted prior to be sequenced in a NextSeq500 High Output run (Illumina) with a 150 cycle single read. Around 3% of the PhiX control was mixed with the pool for sequencing. Fastq files were generated using bcl2fastq Software (Illumina).

Regarding the data analysis, the raw FASTQ files were trimmed and filtered with Trimmomatic v0.36 ^52^ using the single end approach with the following flags: “LEADING:3 TRAILING:3 SLIDINGWINDOW:4:15 MINLEN:75”. The trimmed FASTQ files were converted to FASTA with the command “fastq_to_fasta” from FASTX-Toolkit v0.0.14.^53^ From these generated FASTA files, spacers were extracted by following two different approaches.

Spacer extraction and analysis was performed as previously described.^27^ Briefly, spacers were considered to be the genomic regions of 20–66 nucleotides delimited by DR1 (GAATTGAAAC) and DR2 (GTCGTACTTT), allowing for 2 and 3 mismatches in DR1 and DR2, respectively. Only unique spacers (>1 mismatch) for each sample were processed further. These genetic fragments were extracted and aligned to the DFL 12 reference genome (GenBank CP000830) and plasmid with BWA v0.7.17.^54^ The spacer length distributions were plotted with a Python custom script (v3.10.12). Once aligned, the output SAM files were processed to remove duplicates. Tables of counts were obtained with the FeatureCounts implementation of Subread v.1.5.1.^55^ This restrictive approach yielded very sparse counts for the genome of and prevented further differential spacer acquisition analysis under the studied conditions. As a second approach, spacers were extracted with a custom Python script that did not restrict spacers by nucleotide length, but conserved the allowed mismatches from the first approach. To study differences in spacer acquisitions between samples, count data were analyzed with custom R scripts (v4.1.2). Genes with less than 10 counts across conditions were discarded. For the genome counts, a variance stabilizing transformation was applied, and the data were visualized with the plotPCA function. The differential acquisition analysis was performed with DESeq2 v.1.34 ^55^ for the plasmid counts using standard parameters. Relative spacer count was calculated with CPM-transformed plasmid counts for FbFp sensor divided by total spacers for each sample and plotted with a custom Python script.

## Supplementary Material







## Data Availability

Plasmids used in this study are listed in Table S1, and full sequences are available in File S2. Relevant plasmids will be available in Addgene. All sequencing reads from RNA-seq and recording experiments have been deposited in the European Nucleotide Archive (ENA) repository under PRJEB90807 accession number.

## Notes

A table with the plasmids used in this work accompanied by a short description; representative SEM images of biofilms and TEM images of bacterial cells showing more extensive expression of curli fibers; representative TEM images showing how the percentage of curli-producing cells was quantified; assessment of biofilm integrity following centrifugal force application; RT-qPCR amplification plot for *melC2* gene in *D. shibae* tyrosinase producing strain; western blot analysis of wild-type and tyrosinase-producing strain; crystal violet assay to determine the optimal ratio of tyrosinase to dsCsgA-Mfp3 variants; characterization of FbFP fluorescent signal of the temperature biosensor strain within an actual biofilm in a 96 well plate; principal component analysis (PCA) of the genome aligned spacers of control and heat-shock treated samples; hierarchical clustering of the genome counts by sample treatment (PDF)
The data of the differential expression for the different genes of after 42 °C heat shock compared with a control (XLSX)
Additional details of the plasmid sequences in GenBank format (ZIP)
