# Supplementary material for: Engineered Marine Biofilms for Ocean Environment Monitoring
Source: ACS Synth Biol. 2025 Jun 23;14(7):2797–809. doi: 10.1021/acssynbio.5c00192 (PMC12281610; doi:10.1021/acssynbio.5c00192)

Supporting Information

Engineered marine biofilms for ocean environment monitoring

Guillermo Nevot[1,*], Maria Pol Cros[1,*], Lorena Toloza[1], Nil Campamà-Sanz[1,3], Maria Artigues-Lleixà[1], Laura Aguilera[1] and Marc Güell[1, 2,+]

*These authors contributed equally
+correspondence to Marc Güell (marc.guell@upf.edu)

This PDF file includes:

Supporting tables

- Supporting Table S1.- Plasmids used in this study.

Supporting figures

- Supporting Figure S1.- SEM and TEM imaging of *D. shibae* curli fibers.
- Supporting Figure S2.- Assessment of biofilm integrity following centrifugal force application.
- Supporting Figure S3.- RT-qPCR amplification plot for *melC2.*
- Supporting Figure S4.- Western blot analysis of *D. shibae* wild type (WT) and tyrosinase-producing (Tyr) strain.
- Supporting Figure S5.- Crystal Violet assay to determine the optimal ratio of *D. shibae* tyrosinase to *D. shibae* dsCsgA-Mfp3 variants.
- Supporting Figure S6.- Characterization of FbFP fluorescent signal of the temperature biosensor *D. shibae* strain within an actual biofilm.
- Supporting Figure S7.- PCA and hierarchical clustering of acquired spacers.

This study also provides supporting files including:

- Supporting File S1.- Differential expression of *D. shibae* after 42ºC heat shock compared with a control.
- Supporting File S2.- Plasmid sequences in genbank format.

Supporting Tables

Supporting Table S1.- Plasmids used in this study.

Plasmid name	Description	Reference
pBBR1MCS-2	Broad-host range vector with pBBR1 origin of replication and Cm^R cassette and oriT.	Piekarski et al. 2009
pDS_P(aphII)-dsCsgA-Mfp3-A	Plasmid for dsCsgA-Mfp3A expression with the *aphII* promoter upstream of the endogenous *dsCsgA* promoter.	This study
pDS_P(dsCsgA)-dsCsgA-Mfp3-A	Plasmid for dsCsgA-Mfp3A expression with the endogenous *dsCsgA* promoter.	This study
pDS_P(aphII)-dsCsgA-Mfp3-S	Plasmid for dsCsgA-Mfp3-S expression with the *aphII* promoter upstream of the endogenous *dscsgA* promoter.	This study
pDS_P(dsCsgA)-dsCsgA-Mfp3-S	Plasmid for dsCsgA-Mfp3-S expression with the endogenous *dsCsgA* promoter.	This study
pDS_P(aphII)-dsCsgA-Mfp3-SP	Plasmid for dsCsgA-Mfp3-SP expression with the *aphII* promoter upstream of the endogenous *dsCsgA* promoter.	This study
pDS_P(dsCsgA)-dsCsgA-Mfp3-SP	Plasmid for dsCsgA-Mfp3-SP expression with the endogenous *dsCsgA* promoter.	This study
pDS_Tyrosinase	Plasmid encoding OsmY-MelC2 fusion proteinwith the *aphII* promoter for tyrosinase activity.	This study
pDS_Temp	Plasmid encoding FbFP controlled by the temperature sensitive promoter derived from Dshi_0075.	This study
pDS_OxyA	Plasmid encoding FbFP controlled by the oxygen sensitive promoter derived from *hemN2* (Dshi_0659).	This study
pDS_OxyB	Plasmid encoding FbFP controlled by the oxygen sensitive promoter derived from *hemN2* (Dshi_0659) and dsFnrL controlled by the *aphII* constitutive promoter.	This study
pDS_Recording	Plasmid encoding the RT-Cas1 and Cas2 proteins controlled by the *aphII* constitutive promoter and the CRISPR array from *Fusicatenibacter saccharivorans.*	This study

Plasmid name	Description	Reference
pDS_TempRecording	Same as pDS_Recording but also encoding Fbfp controlled by the temperature sensitive promoter from Dshi_0075.	This study

Supporting Figures

Supporting Figure S1.- (A) SEM images of *D. shibae* wild type strain (WT) and *D. shibae* Mfp3-A engineered variants with two different promoters, P(aphII) and P(dsCsgA), respectively, showing an extensive curli fiber network in the P(dsCsgA)-dsCsgA-Mfp3-A variant. Images were captured at a magnification of 9,500x. (B) TEM images of *D. shibae* WT bacterial cells and *D. shibae* Mfp3-A engineered variants, showing more extensive expression of curli fibers extending from the plasma membrane in the Mfp3-A variants. Images were captured at a magnification of 60,000x. (C) TEM images of the same *D. shibae* strains, captured at a magnification of 20,000x. (D) An example of how the percentage of curli-producing cells was quantified, with curli-producing bacteria circled in purple.

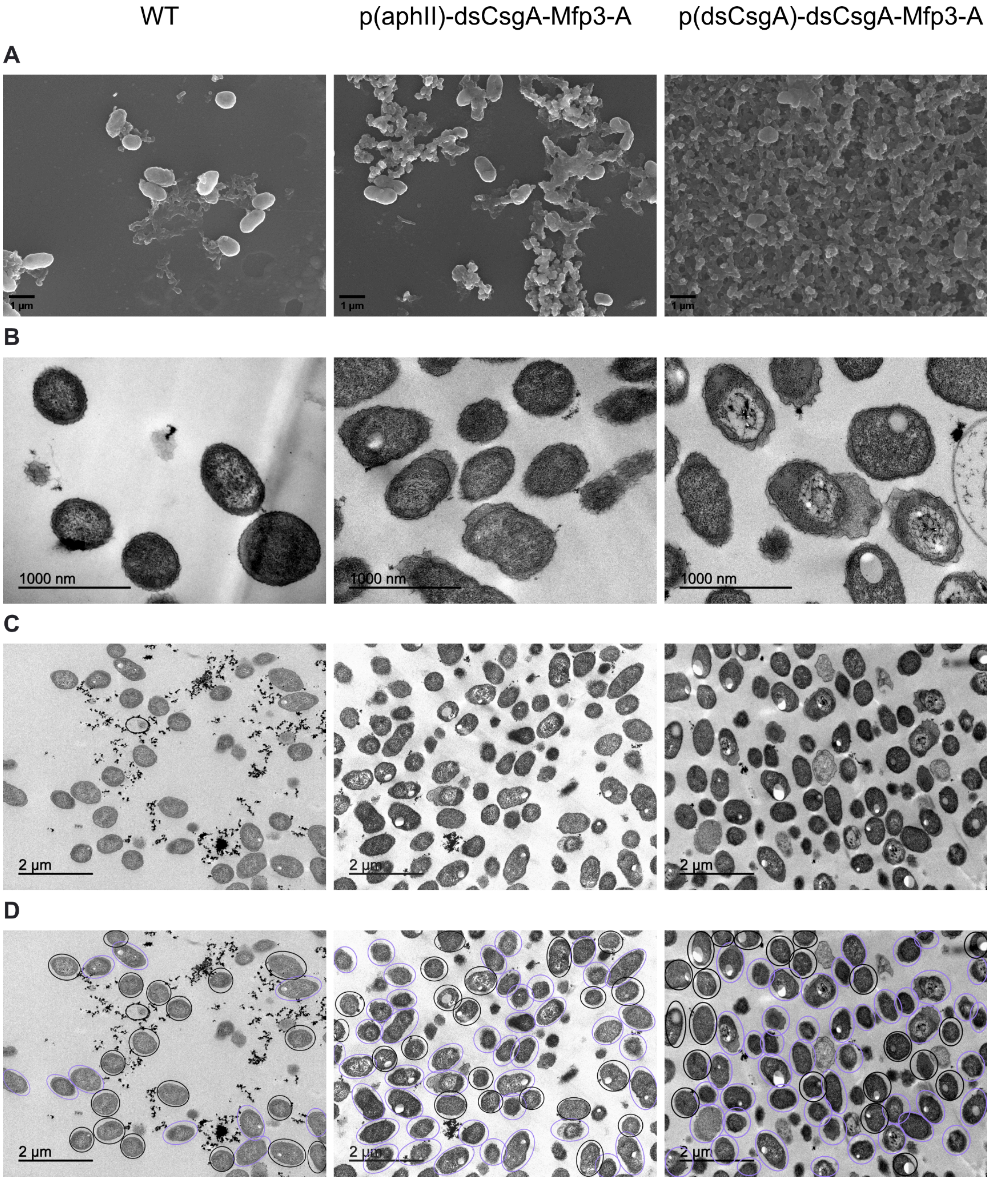

Supporting Figure S2.- Assessment of biofilm integrity following centrifugal force application. Biofilm loss was quantified by comparing the A_{590nm}/OD_{600nm}. ratio of centrifuged and inverted plates to the control. The *D. shibae* P(dsCsgA)-dsCsgA-Mfp3A variant exhibited less biofilm loss than the empty plasmid control. Data are presented as mean ± SD (*$p \leq 0.05$, **$p \leq 0.01$, ***$p \leq 0.001$).

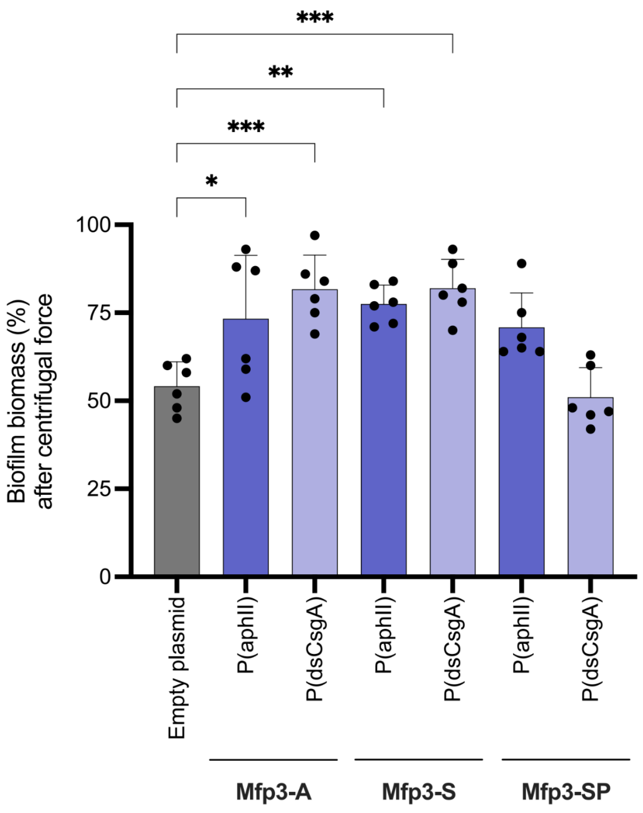

Supporting Figure S3.- RT-qPCR amplification plot showing positive mRNA expression of the *melC2* gene in *D. shibae* tyrosinase producing strain (Ct ≈ 18).

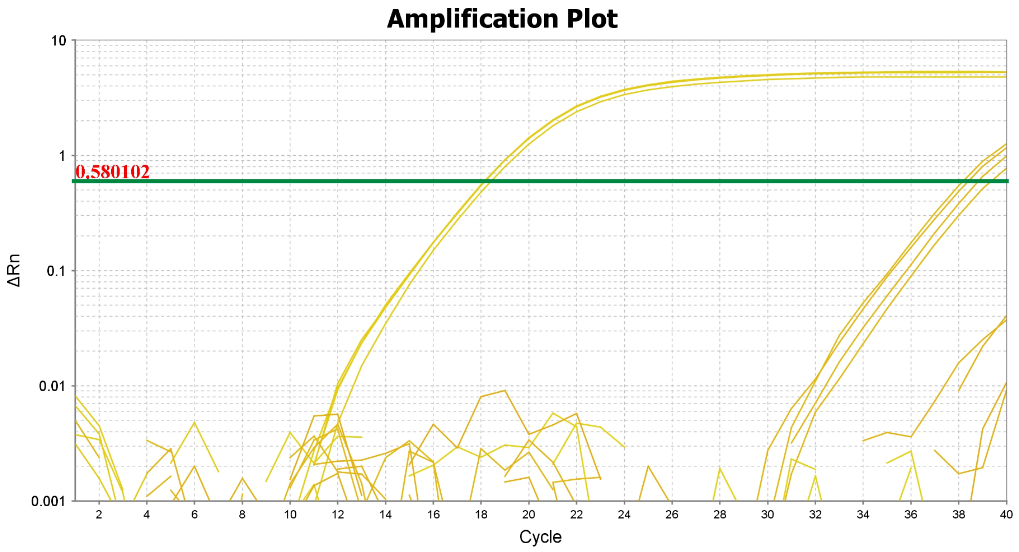

Supporting Figure S4.- Western blot analysis of *D. shibae* wild type (WT) and tyrosinase-producing (Tyr) strain. Lanes represent the supernatant, periplasmic fraction, and cytoplasmic fraction, respectively.

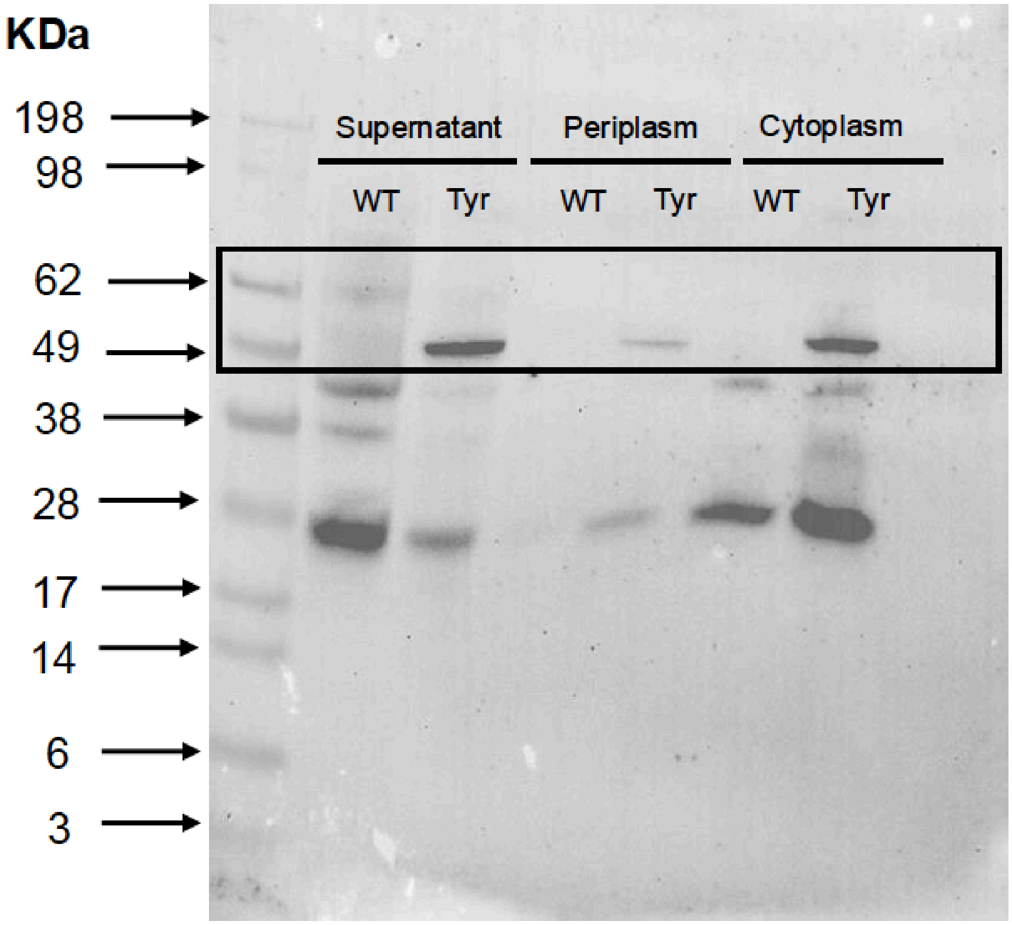

Supporting Figure S5.- Crystal Violet assay to determine the optimal ratio of *D. shibae* tyrosinase to *D. shibae* P(aphII)-Mfp3-SP. Data are presented as mean ± standard deviation (SD) (*p ≤ 0.05, **p ≤ 0.01, ***p ≤ 0.001).

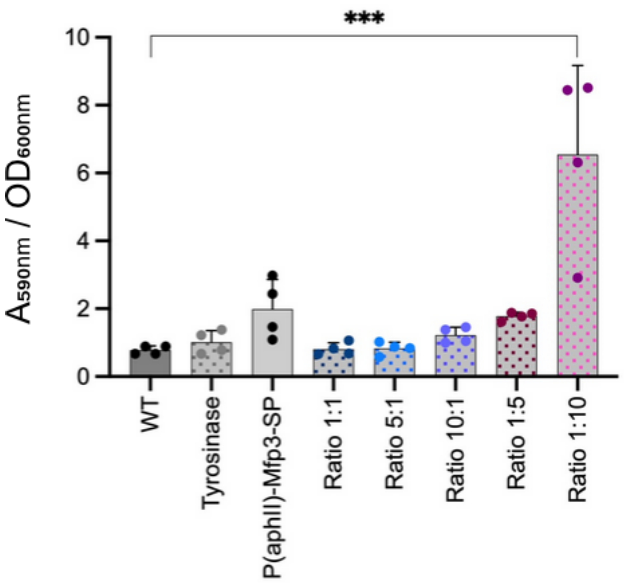

Supporting Figure S6.- FbFP fluorescent signal of the temperature biosensor *D. shibae* strain within an actual biofilm in a 96 well plate after different lengths of heat sock treatment and removal of planktonic bacteria. Fluorescent values are normalized with the OD_{600nm} before the removal of planktonic bacteria.

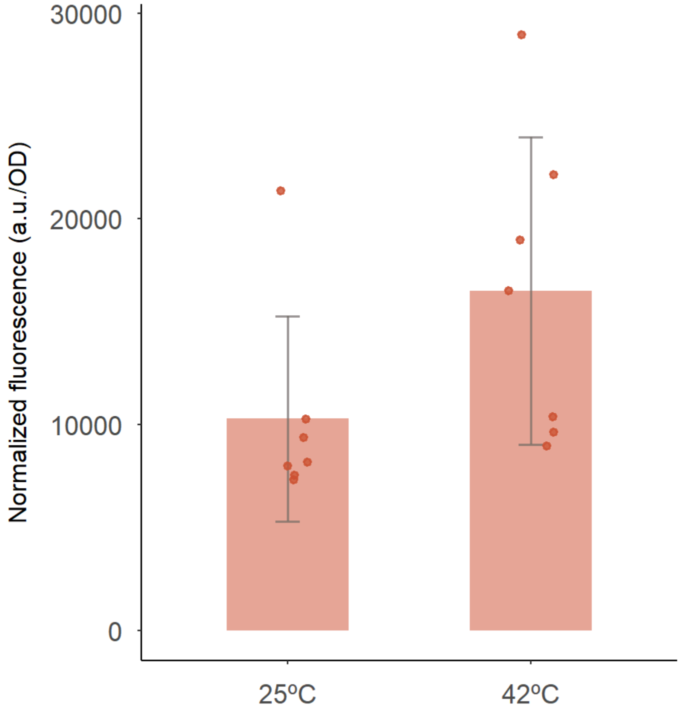

Supporting Figure S7.- (A) Principal Component Analysis of the genome aligned spacers of control and heat-shock treated samples. (B) Hierarchical Clustering of the genome counts by sample treatment.

A

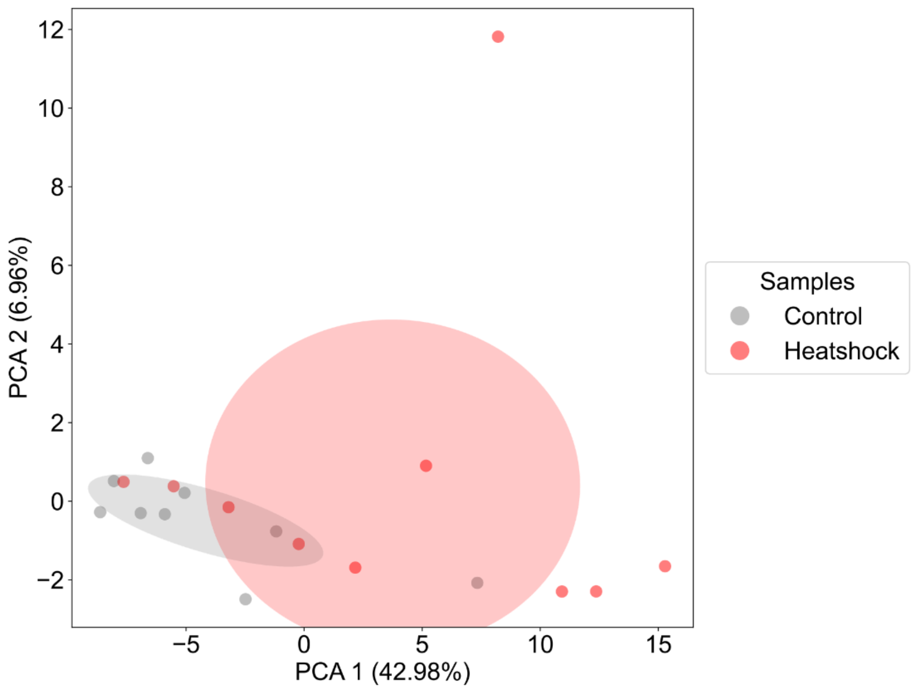

B

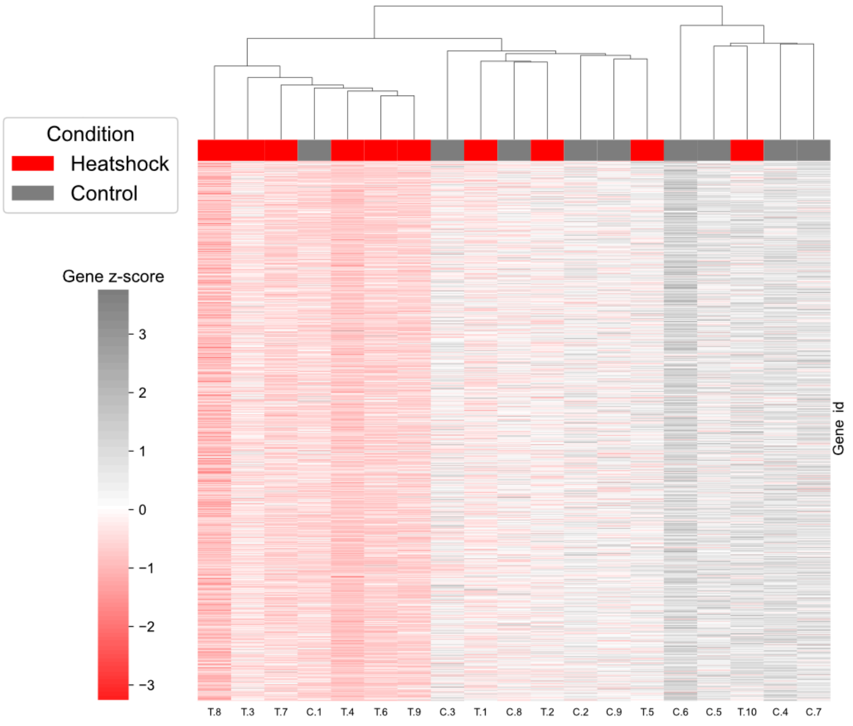

Supplement: Supplementary file 1 [file sb5c00192_si_001.pdf]
